# Supplementary material for: Sex differences in learning from exploration
Source: eLife. 2021 Nov 19;10:e69748. doi: 10.7554/eLife.69748 (PMC8794469; doi:10.7554/eLife.69748)
Supplement: Supplementary file 1. [file elife-69748-supp1.docx]

**Supplemental Table 1: Four-way Repeated Measures ANOVA**

| **Effect** | **Sum Sq.** | **df.** | **Mean Sq.** | | **F** | **p value** |
| --- | --- | --- | --- | --- | --- | --- |
| sex | 0.0782 | 1 | 0.0782 | 6.89 | | 0.0088 |
| subject(sex) | 2.1709 | 30 | 0.0904 | 7.95 | | < 0.00001 |
| reward | 13.1509 | 1 | 13.1509 | 1157.63 | | < 0.00001 |
| state | 35.4862 | 1 | 35.4862 | 3123.74 | | < 0.00001 |
| sex x reward | 0.2094 | 1 | 0.2094 | 18.44 | | < 0.00001 |
| sex x state | 0.0383 | 1 | 0.0383 | 3.37 | | 0.0667 |
| subject(sex) x reward | 3.616 | 30 | 0.1205 | 10.61 | | < 0.00001 |
| subject(sex) x state | 1.8529 | 30 | 0.0618 | 5.44 | | < 0.00001 |
| reward x state | 0.4652 | 1 | 0.4652 | 40.95 | | < 0.00001 |
| sex x reward x state | 0.0463 | 1 | 0.0463 | 4.07 | | 0.0438 |
| subject(sex) x reward x state | 0.5573 | 30 | 0.0186 | 1.64 | | 0.0177 |
| error | 9.872 | 869 | 0.0114 |  | |  |
| total | 68.5207 | 996 |  |  | |  |
