# Supplementary material for: Sex differences in learning from exploration
Source: eLife. 2021 Nov 19;10:e69748. doi: 10.7554/eLife.69748 (PMC8794469; doi:10.7554/eLife.69748)
Supplement: Supplementary file 2. [file elife-69748-supp2.docx]

**Supplemental Table 2: Tukey’s multiple comparison test of model agreement across RL models**

| **Model** | **Mean Diff.** | **95% CI of diff.** | **Adjusted p value** |  |
| --- | --- | --- | --- | --- |
| random vs. noisy WSLS | 0.058 | 0.0120 to 0.096 | 0.0008 | *** |
| random vs. RL | -0.073 | -0.095 to -0.052 | <0.0001 | **** |
| random vs. RLε | -0.074 | -0.093 to -0.054 | <0.0001 | **** |
| random vs. RLCK | -0.096 | -0.116 to -0.076 | <0.0001 | **** |
| random vs. RLCKγ | -0.100 | -0.119 to -0.080 | <0.0001 | **** |
| random vs. RLCKη | -0.098 | -0.118 to -0.078 | <0.0001 | **** |
| noisy WSLS vs. RL | -0.131 | -0.168 to -0.095 | <0.0001 | **** |
| noisy WSLS vs. RLε | -0.131 | -0.168 to -0.095 | <0.0001 | **** |
| noisy WSLS vs. RLCK | -0.154 | -0.191 to -0.116 | <0.0001 | **** |
| noisy WSLS vs. RLCKγ | -0.157 | -0.193 to -0.122 | <0.0001 | **** |
| noisy WSLS vs. RLCKη | -0.156 | -0.193 to -0.119 | <0.0001 | **** |
| RL vs. RLε | 0.000 | -0.009 to 0.009 | >0.9999 | ns |
| RL vs. RLCK | -0.023 | -0.038 to -0.013 | <0.0001 | **** |
| RL vs. RLCKγ | -0.026 | -0.035 to -0.017 | <0.0001 | **** |
| RL vs. RLCKη | -0.025 | -0.034 to -0.01533 | <0.0001 | **** |
| RLε vs. RLCK | -0.022 | -0.031 to -0.013 | <0.0001 | **** |
| RLε vs. RLCKγ | -0.026 | -0.034 to -0.018 | <0.0001 | **** |
| RLε vs. RLCKη | -0.024 | -0.034 to -0.014 | <0.0001 | **** |
| RLCK vs. RLCKγ | -0.004 | -0.011 to 0.003 | 0.675 | ns |
| RLCK vs. RLCKη | -0.002 | -0.010 to 0.006 | 0.9914 | ns |
| RLCKγ vs. RLCKη | 0.002 | -0.007 to 0.010 | 0.9951 | ns |
